# Social coevolution and Sine chaotic opposition learning Chimp Optimization Algorithm for feature selection

**DOI:** 10.1038/s41598-024-66285-6

**Published:** 2024-07-04

**Authors:** Li Zhang, XiaoBo Chen

**Affiliations:** 1 College of Computer Engineering, Jiangsu University of Technology, Changzhou, 213001 People’s Republic of China; 2 Key Laboratory of Symbolic Computation and Knowledge Engineering of Ministry of Education, Jilin University, Changchun, 130012 People’s Republic of China; 3 People’s Bank of China Changzhou City Center Branch, Jiangsu, 213001 Changzhou People’s Republic of China

**Keywords:** Chimp optimization algorithm, Feature selection, High-dimensional classification data, Social coevolution, Sine chaotic opposition learning, Computational science, Information technology, Scientific data

## Abstract

Feature selection is a hot problem in machine learning. Swarm intelligence algorithms play an essential role in feature selection due to their excellent optimisation ability. The Chimp Optimisation Algorithm (CHoA) is a new type of swarm intelligence algorithm. It has quickly won widespread attention in the academic community due to its fast convergence speed and easy implementation. However, CHoA has specific challenges in balancing local and global search, limiting its optimisation accuracy and leading to premature convergence, thus affecting the algorithm’s performance on feature selection tasks. This study proposes Social coevolution and Sine chaotic opposition learning Chimp Optimization Algorithm (SOSCHoA). SOSCHoA enhances inter-population interaction through social coevolution, improving local search. Additionally, it introduces sine chaotic opposition learning to increase population diversity and prevent local optima. Extensive experiments on 12 high-dimensional classification datasets demonstrate that SOSCHoA outperforms existing algorithms in classification accuracy, convergence, and stability. Although SOSCHoA shows advantages in handling high-dimensional datasets, there is room for future research and optimization, particularly concerning feature dimensionality reduction.

## Introduction

In the last few decades, new computer and Internet technologies have generated large amounts of high-dimensional data at an accelerated rate as never before^1,2^. There are many irrelevant and redundant features contained in these high-dimensional data. Because irrelevant and redundant features not only increase the training time of the model but also make the model poorly interpretable^3^. Dealing with these irrelevant and redundant features is a significant challenge in machine learning and data mining^4^. Feature selection^5^ differs from other data dimensionality reduction techniques (e.g. feature extraction) in that it removes irrelevant and redundant features and retains relevant original physical features, thereby reducing the dimensionality of the data. This helps to improve the data quality and classification performance and allows the training time of the model to be significantly reduced and the model to become more interpretable. It has, therefore, attracted the attention of many researchers^6^.

Exhaustive search in a high-dimensional data space is completely impractical^7^. Swarm intelligence algorithms^8^ allow the best solution to be selected from a set of available alternatives by constructing an appropriate fitness function to obtain a globally optimal solution. For example, the Bat Algorithm (BA)^9^, the Flower Pollination Algorithm (FPA)^10^, the Grey Wolf Optimization Algorithm (GWO)^11^,the Whale Optimization Algorithm (WOA)^12^, Harris Hawk Optimization Algorithm (HHO)^13^, Chimp Optimization Algorithm (CHoA)^14^, Butterfly Optimization Algorithm (BOA)^15^, etc. These algorithms use the “trial and error” principle in finding the optimal solution and can be successfully applied to solve global optimization problems.

Like other swarm intelligence algorithms, CHoA faces some challenging issues, such as the balance between exploration and exploitation, the improvement of the quality of the solution, and the avoidance of falling into local optima. These issues impact the classification accuracy and convergence speed of the CHoA algorithm. The “No Free Lunch” theorem^16^ states that no universal optimisation technique can solve all optimisation problems. This theorem has contributed to the active development of intelligent optimisation, prompting the improvement of existing algorithms and the proposal of new methods. Therefore, this paper improves the CHoA algorithm and proposes a Chimp optimization algorithm called Social coevolution and Sine Chaos oppositional Learning (SOSCHoA). It has been proved experimentally that SOSCHoA performs excellently in solving the feature selection problem.

The CHoA algorithm can be divided into updating and finding the global optimum. This paper introduces two operators to improve the algorithm, enhance classification accuracy and accelerate the convergence speed. The main contributions of this paper are summarized as follows:

- The social coevolutionary strategy was utilised in the location update equation to promote information sharing among individuals within the chimp population. To enhance the algorithm exploration ability and maintain algorithm diversity while effectively balancing the ability between local exploration and global exploitation.
- Sine chaotic opposition learning strategy is introduced in the stage of finding the global optimal solution. This strategy increases the randomness and diversity of the chimp population’s search direction and the possibility of the algorithm finding the global optimal solution. Finally, the greedy principle is used to select the current optimal solution among the Sine Chaos population solution and the original solution to speed up the algorithm’s convergence.
- The SOSCHoA algorithm was evaluated for comprehensive performance using 12 high-dimensional classification datasets. The experimental results show that the SOSCHoA algorithm outperforms other algorithms, including (CHoA^14^, the DLF for improving the performance of ChOA (DLFCHOA)^17^, the lens-imaging based learning Butterfly Optimization Algorithm (PIL-BOA)^18^, A new variant of the BOA (BBOA)^19^, improved variant of the arithmetic optimization algorithm (LMRAOA)^20^, velocity-guided Harris hawks optimizer (VGHHO)^21^, FA^22^, FPA^10^, WOA^12^, HHO^13^, Manta Ray Foraging Optimization (MRFO)^23^).

The remainder of this paper is specified below. Section “Related work” reviews related work. Section “Chimp Optimization Algorithm” discusses the CHoA algorithm. An improved Chimp optimization algorithm is presented in “Proposed improved chimp optimization algorithm”. “Description of SOSCHoA algorithm” presents the SOSCHoA feature selection algorithm and the pseudo-code implementation. In “Experimental validation and analysis”, an experimental study is conducted, and the results are discussed. “Conclusion” concludes the research and presents ideas for further work.

## Related work

High-dimensional optimization problems are widely found in engineering applications and scientific computing, for example, wind turbine fleet optimization^24^ and automobile side impact optimization^25^. However, Swarm intelligence algorithms mainly suffer from poor solution quality and the tendency to fall into local optima in high-dimensional optimization problems. Therefore, many researchers have proposed improvement strategies in many aspects to avoid falling into local optima and finding globally optimal solutions. Therefore, many researchers have proposed improvement strategies to avoid falling into local optimum and accelerate the convergence speed. Table 1 lists some Swarm intelligence algorithms for solving high-dimensional optimization problems.

**Table 1:** Research on meta-heuristic algorithms for high-dimensional data.

Algorithm	Authors	Brief description
ISSAFD	Neggaz^26^	Salp swarm Algorithm, Sine Cosine algorithm and Disrupt Operator
CLBCSA	Braik^27^	Chaotic sequence and Lévy flight
SCHHO	Hussain^28^	Sine-cosine algorithm and Harris hawks optimization
BChOA	Wang^29^	V-shaped functions and S-shaped functions
BIGWO	Rajalaxmi^30^	V-shaped functions and S-shaped functions
iBSSA	Gad^31^	V-shaped functions and S-shaped functions
ABGWO	Wang^32^	Adaptive coefficients and S-shaped functions
LIL-HHO	Long^33^	Lens imaging learning
SChoA	Kaur^34^	Sine cosine function
BGEO-IFNS	Yang^35^	Binary Golden Eagle Optimizer and Initialization of Feature Number Subspace
E-WOA	Nadimi-Shahraki^36^	pooling mechanism, migrating, preferential selecting, and enriched encircling prey
BCHoA-C	Pashaei^37^	Fusion of MRMR and CHoA algorithms
NChOA	Gong^38^	niching technology
EChOA	Jia^39^	Spearman’s rank correlation coefficient and the beetle antennae operator
ULChOA	Liu^40^	best knowledge
AChOA	Wang^41^	Tent chaotic map, adaptive weight and the Lévy flight strategy
ECH3OA	Fahmy^42^	HHO and CHoA
MFO-SFR	Nadimi-Shahraki^43^	stagnation finding and replacing strategy
EHHO	Peng^44^	Hierarchies and V-functions
VGHHO	Long^21^	Refraction of opposing learning mechanisms
MSGWO	Chang^45^	Random Opposition-based Learning, Non-linear adjustment and two-stage hybrid mutation operator
mSTOA	Houssein^46^	balancing exploration/exploitation strategy
cHGWOSCA	Duan and Yu^47^	Fusion of SCA and GWO algorithms
GSOBL-ChOA	Bo^48^	Greedy choices and oppositional learning

Neggaz^26^ proposed ISSAFD, which relies on the use of the sine cosine algorithm and perturbation operators to improve the performance of the slap swarm algorithm.Hussain^28^ proposed the SCHHO algorithm, which enhances the development by fusing the sine-cosine algorithm and Harris Hawks optimisation to dynamically adjust the candidate solutions to avoid the problem of solution stagnation in HHO.Braik^27^ proposed Chaotic sequence and Lévy flight with BCSA (CLBCSA).CLBCSA combines chaotic sequences and Lévy flights to enhance the algorithm’s local exploitation capabilities while maintaining its global search capabilities. This combination strategy aims to improve the algorithm’s ability to avoid falling into local optima and to converge quickly to the global optimum. Yang^35^ proposed the Binary Golden Eagle Optimizer algorithm combined with the Initialization of Feature Number Subspace (BGEO-IFNS). With the IFNS approach, BGEO-IFNS can initially generate higher-quality populations, improving the algorithm’s ability to search in a high-dimensional search space and the final optimisation performance.Nadimi-Shahraki^36^ proposed the E-WOA algorithm, which solves the feature selection problem using a pooling mechanism and three effective search strategies. Finally, the E-WOA algorithm was applied to COVID-19 disease feature selection. Rajalaxmi^30^ proposed the BIGWO algorithm. First, the optimal solution is solved by the GWO algorithm; then, the optimal subset of features is obtained by binary conversion of the optimal solution with V- and S-shaped functions. Gad^31^ proposed the iBSSA algorithm, firstly, to improve the local exploration capability using a local search algorithm; secondly, to improve the global search capability using a roaming agent approach; and finally, to obtain the optimal feature subset by a binary transformation of the optimal solution using V- and S-shaped functions. Wang^32^ proposed the ABGWO algorithm. First, an adaptive coefficient is introduced to improve the local exploration capability and global search capability of the GWO algorithm. Finally, the optimal feature subset is obtained by binary conversion of the optimal solution by a Sigmoid transformation function.Long^33^ proposed LIL-HHO. First, the escape energy E is improved by a sinusoidal function to achieve a good transition from the exploration phase to the exploitation phase. Second, the search accuracy is enhanced by introducing the individual’s best position for each eagle. Third, crystal imaging learning is used to eliminate the local optimum and thus obtain the global optimum solution. Finally, experiments prove that this algorithm outperforms the comparison algorithm. Peng^44^ proposed the EHHO algorithm. First, the optimal solution is obtained by optimizing the HHO algorithm through a hierarchical structure. Then, the optimal subset of features is obtained by binary conversion of the optimal solution through a V-transformation function. Chang^45^ proposed by the MSGWO algorithm. First, a Random Opposition-based Learning (ROL) strategy is applied to improve the population quality in the initialisation phase. Secondly, the convergence factor is adjusted nonlinearly to reconcile global exploration and local exploitation capabilities. Finally, a two-stage mixed-variance operator is introduced to increase population diversity and balance the exploration and exploitation capabilities of GWO. Houssein^46^ proposed the mSTOA algorithm. The algorithm uses a balanced exploration/exploitation strategy, an adaptive control parameter strategy, and a population reduction strategy to solve the problem of poor convergence and improve classification accuracy. Duan^47^ proposed the cHGWOSCA algorithm. First, the SCA algorithm is used to update the position of the head wolf; second, the grey wolf is guided to search for prey using moderate value weights and individual optimal positions to obtain the global optimal solution.Nadimi-Shahraki^43^ proposed by the MFO-SFR algorithm improves the performance of the search process through the stagnation finding and replacing (SFR) strategy. Secondly, archives are used to enrich the diversity of the population. Finally, experiments prove that the algorithm is effective.

Wang^29^ proposed the BChOA algorithm. First, the optimal solution is found by the ChOA algorithm. Then, the optimal subset of features is obtained by binary transformation of the optimal solutions by V- and S-type functions.Pashaei^37^ proposed the BCHoA-C algorithm. Firstly, the MRMR algorithm ranks the feature set and filters a subset of features with high relevance and low redundancy. Secondly, the CHoA algorithm finds the optimal solution. Finally, the optimal subset of features is obtained by binary conversion of the optimal solution using V-type and Sigmoid conversion functions. Khishe^49^ proposed OBLChOA. This algorithm gets the global optimal solution using a greedy search and backward learning strategy. Jia^39^ suggested EChOA, which firstly initializes the population using polynomial mutation; secondly, calculates the gap between the lowest social status chimp and the leader chimp via Spearman’s rank correlation coefficient; and finally, uses the beetle’s tentacle operator to jump out of the local optimum to obtain the global optimum solution. Liu^40^ proposed ULChOA, an algorithm that updates the location of prey using a generic learning mechanism that provides a dynamic balance between the exploration and exploitation phases. The algorithm was finally demonstrated to be effective through experiments. Kaur^34^ proposed the SChoA algorithm. The algorithm solves the slow convergence by improving the Chimp’s search and updating the equation with a sine cosine function to obtain the optimal solution. Gong^38^ proposed the NChOA algorithm, which uses niching techniques, individual optimal techniques for PSO, and local search techniques to improve search efficiency and increase convergence speed. Wang^41^ proposed AChOA, initialising the population through a Tent chaotic mapping. Secondly, it uses an adaptive non-linear convergence factor and adaptive weight coefficients to improve population diversity. Finally, a Lévy flight strategy is applied to jump out of the local optimum. The method is experimentally proven to be effective. Fahmy^42^ proposed ECH3OA, which obtains the global optimal solution by combining a fusion of the enhanced Chimp Optimization Algorithm (ChOA) and Harris Hawkes Optimization Algorithm (HHO). Bo^48^ proposed the GSOBL-ChOA algorithm. Firstly, the convergence rate is accelerated by applying the OBL technique in the exploration phase of ChOA. Second, a greedy selection strategy is used to find the optimal solution.

Although the swarm intelligence algorithms mentioned above improve search efficiency and increase convergence speed, they still suffer from unbalanced exploration and exploitation, poor solution quality, and tend to fall into local optimality. According to our study, enhancing local exploration, increasing population diversity, and finding globally optimal solutions have become essential for studying swarm intelligence algorithms in high-dimensional optimization^50–52^. Therefore, this paper focuses on the location update equations and global optimization mechanisms in the CHoA algorithm. It proposes a Chimp optimization algorithm with a coevolutionary strategy and Sine chaotic opposition learning and also applies it to the high-dimensional classification feature selection problem.

## Chimp Optimization Algorithm

The CHoA algorithm is a swarm intelligence optimization algorithm proposed to simulate the hunting behaviour of a chimp in nature. The chimp hunting process is generally divided into chasing and attacking the prey. The standard CHoA algorithm selects an attacker (first optimal solution), a barrier (second optimal solution), a chaser (third optimal solution), and a driver (fourth optimal solution) to discover potential prey locations jointly. In the search spaces, the chimp group mainly uses the four best-performing chimps to guide the other chimps toward their optimal areas, while the four chimps - attacker, barrier, chaser, and driver - predict the possible locations of the captured objects during the continuous iterative search by guiding the continuous search for the global optimal solution. The mathematical model of a chimp chasing prey during the search process is, therefore, as follows:

1 $$\begin{aligned} {X_{chimp}}\left( {t + 1} \right) = {X_{prey}}\left( t \right) - a \cdot \left| {C \cdot {X_{prey}}\left( t \right) - m \cdot {X_{chimp}}\left( t \right) } \right| \end{aligned}$$

In Eq. (1), ${X_{prey}}$the position vector of the prey, ${X_{chimp}}$ the position vector of the current individual chimp, *t* the number of current iterations, and *a*, *C*, *m* the coefficient vector, which is calculated as follows:

2 $$\begin{aligned} a= & {} 2 \cdot f \cdot {r_1} - f \end{aligned}$$

3 $$\begin{aligned} C= & {} 2 \cdot {r_2} \end{aligned}$$

4 $$\begin{aligned} m= & {} Chaotic\_value \end{aligned}$$

5 $$\begin{aligned} f= & {} 2.5 - \frac{{2.5 \cdot t}}{{{t_{\max }}}} \end{aligned}$$

Among them, ${r_1}$ and ${r_2}$ are random numbers between $\left[ {0,1} \right]$, respectively. *f* is the convergence factor whose value decreases non-linearly from 2.5 to 0 as the number of iterations increases. ${t_{\max }}$ is denoted as the maximum number of iterations. *a* is a random vector that determines the distance between the chimp and the prey, with a random number of values between $\left[ { - f,f} \right]$ .*C* is the chaotic vector generated by the chaotic mapping. *C* is the control coefficient for the Chimp expulsion and prey chasing, and its value is a random number between $\left[ {0,2} \right]$.

The mathematical model for the chimp attack on prey is as follows:

6 $$\begin{aligned} {X_1}= & {} {X_{attacker }} - {a_1} \cdot \left| {{C_1} \cdot {X_{attacker }} - {m_1} \cdot X\left( t \right) } \right| \end{aligned}$$

7 $$\begin{aligned} {X_2}= & {} {X_{barrier}} - {a_2} \cdot \left| {{C_2} \cdot {X_{barrier}} - {m_2} \cdot X\left( t \right) } \right| \end{aligned}$$

8 $$\begin{aligned} {X_3}= & {} {X_{chaser}} - {a_3} \cdot \left| {{C_3} \cdot {X_{chaser}} - {m_3} \cdot X\left( t \right) } \right| \end{aligned}$$

9 $$\begin{aligned} {X_4}= & {} {X_{driver}} - {a_4} \cdot \left| {{C_4} \cdot {X_{driver}} - {m_4} \cdot X\left( t \right) } \right| \end{aligned}$$

10 $$\begin{aligned} {X_{chimp}}\left( {t + 1} \right)= & {} {{\left( {{X_1} + {X_2} + {X_3} + {X_4}} \right) }/ 4} \end{aligned}$$

11 $$\begin{aligned} {X_{chimp}}\left( {t + 1} \right)= & {} \left\{ {\begin{array}{*{20}{c}} {Eq.\left( {10} \right) ,\mu < 0.5}\\ {Chaotic\_value,\mu \ge 0.5} \end{array}} \right. \end{aligned}$$

From Eqs. (6) to (11), $X\left( t \right)$ is the position vector of the current Chimp, ${X_{attac\ker }}$ is the position vector of the attacker, ${X_{barrier}}$ is the position vector of the barrier, ${X_{chaser}}$ is the position vector of the chaser, ${X_{driver}}$ is the position vector of the driver and ${X_{chimp}}\left( {t + 1} \right)$ is the updated position vector of the current Chimp. ${X_{chimp}}\left( {t + 1} \right)$ is the chaotic mapping, which is used to update the position of the solution. From Eq. (10), it is clear that individual chimp positions are estimated from the four best individual chimps, while the other Chimps update their positions randomly.

From Eq. (11), it can be seen that to simulate the social behaviour of chimps attacking their prey, let *u* be a random number between $\left[ {0,1} \right]$. When $u < 0.5$, Eq. (10) is used for the position update. When $u \ge 0.5$, location updates using chaotic process mapping were employed, and this approach determined the chimp’s attack behaviour randomly.

## Proposed improved chimp optimization algorithm

The traditional CHoA has several limitations, such as falling into local optima, slow convergence, and imbalanced development. Therefore, our work aims to develop new variants of CHoA. The proposed algorithm does not affect the basic framework of the CHoA algorithm. Still, it only introduces a social coevolution strategy into the CHoA location equation to overcome the blindness of search and dynamically adjust the balance between local exploration and global exploitation. The Sine chaotic opposition learning mechanism improves the full search capability, enabling the algorithm to jump out of the local optimum solution. This is described in detail below.

### Social coevolution strategy

From Eq. (11), individual chimp positions are determined jointly by attackers, barriers, chasers, and drivers or by chaotic process mapping for position updating. This equation update has the following disadvantages:

- When the four key individuals in the population, attackers, barriers, chasers, and drivers, are all caught in a local optimum, the entire population risks tilting towards a locally optimal solution, significantly constraining the algorithm’s global search capability.
- Suppose the attackers, barriers, chasers and drivers, unfortunately, fall into the confines of the local optimal solution during the iterative process. In that case, the whole chimpanzee population may quickly fall into the trap of this local optimum. This severely limits the algorithm’s convergence efficiency and slows its exploration towards the global optimum.
- $Chaotic\_value$, as a randomly generated vector, carries a certain degree of randomness in its triggering mechanism, with about half the probability of being able to be activated. However, this randomness also leads to a need for more stability. Although $Chaotic\_value$ allows individuals to escape from the local optimal solution to a certain extent, it does not fully consider the interactions and information exchanges within the population during the execution of the optimal search. In particular, $Chaotic\_value$ fails to fully utilise the potential of learning and acquiring positional information from other individuals in the population, which somewhat limits its efficacy in improving search efficiency and optimising global solutions.

Therefore, to address the defects in the principle of the above algorithm and to enhance the local exploitation capability of the chimp optimization algorithm and the ability to communicate among chimp individuals, this paper proposes to update the chimp individual positions using a social coevolution strategy with the following equation.

12 $$\begin{aligned} {X_{i + 1}}\left( t \right) = {X_i}\left( t \right) + {r_3} \cdot \left( {{X_{attacker}} - C \cdot R} \right) \end{aligned}$$

In Eq. (12), ${r_3}$ is a random number between $\left[ {0,1} \right]$ . $C = \frac{{{X_i}\left( t \right) + {X_{i - 1}}\left( t \right) }}{2}$ is a co-occurrence quantity, which represents the relationship characteristics of chimp *i* and $i-1$ in the chimp population. *R* is the benefit factor. This representation of the benefit factor *R* allows for an adequate representation of whether individual chimps benefit partially or fully from the interaction. When $R=1$, it means that chimp *i* and chimp $i-1$ gain a small benefit from interacting with each other. When $R=2$, it means that chimp *i* and chimp $i-1$ greatly benefit from interacting with each other.

The ${r_3} \cdot \left( {{X_{attacker}} - C \cdot R} \right)$ is the socially coextensive component, which not only allows the optimal chimp (${X_{attacker}}$) to exchange information with the general chimp but also allows each chimp to exchange information with neighbouring chimp. This approach enables the chimp to no longer search singularly around a circle defined by attackers, barriers, chasers, and drivers. Furthermore, Eq. (12) leads the individual chimp to steadily converge to the optimal value, which improves the algorithm’s search accuracy and speed, obtaining the desired search results.

### Sine chaotic mapping strategy

#### Sine chaotic mapping

Chaos^53^ is a stochastic, non-periodic, and non-convergent approach found in non-linear dynamical systems. In mathematics, chaotic systems are a source of randomness. The main idea is to exploit the random and ergodic nature of chaotic motion by mapping variables into the interval of values in chaotic variables and finally linearly transforming the resulting solution into the space of optimized variables. The standard chaotic mappings in the optimization field are logistic mapping^54^, Tent mapping^55^, etc. Sine chaotic mapping can help the algorithm jump out of the boundaries of local extreme points due to its ability to search in a wide range. Therefore, using this advantage of sine chaotic mapping, the algorithm can explore the solution space more deeply and reduce the risk of falling into sub-optimal solution regions, improving the solution’s quality and the optimisation process’s overall performance^56^. Sine mappings are calculated as follows:

13 $$\begin{aligned} S\left( {x_i^j} \right) = a \cdot \sin \left( {\pi x_i^j} \right) \end{aligned}$$

In equation (13), $a \in \left( {0,1} \right]$ is the control parameter and $S\left( {x_i^j} \right) \in \left[ { - 1,1} \right]$ is the chaotic sequence value.

#### Opposition-based Learning

Opposition-Based Learning (OBL) is a mathematical method proposed by Tizhoosh^57^, the essential principle of which is to select the best solution for the next iteration by estimating and comparing the feasible solution with the inverse solution. Rahnamayan^58^ proposed an opposing learning strategy for the neighbourhood centre of gravity, allowing the particle swarm to take in the group search experience and increasing population diversity. Yin^59^ proposed that introducing adversarial learning competition for local search in the primary particle swarm algorithm can improve the algorithm’s performance in solving high-dimensional optimization. All of these scholars have made it possible for the reverse solution to reach the vicinity of the optimal solution more accurately by using the contrastive learning approach to improve intelligent optimization algorithms. Thus, the computational model of opposing learning is specified as follows:

14 $$\begin{aligned} \overline{x_i^j\mathrm{{ }}} = lb + ub - x_i^j \end{aligned}$$

Among them, ${X_i} = \left\{ {x_i^1,x_i^2, \ldots ,x_i^j} \right\} ,\left( {i = 1,2, \ldots ,N;j = 1,2, \ldots ,D} \right)$ ,*N* is the number of populations and *D* is the dimensional search space. ${X_i}$ is a point in *D* dimensional space. ${X_i}$ is the reverse of $\overline{{X_i}}$.$\overline{x_i^j} \in \left[ {lb,ub} \right]$, $lb = \min \left( {x_i^j} \right)$, $ub = \max \left( {x_i^j} \right)$.

#### Sine chaotic oppositional learning

From the ChoA algorithm description^14^, in performing global exploration, the Chimp algorithm first updates the dimensional information of the solution. Subsequently, it evaluates the fit of the objective function. Next, the fitness value of the current position is compared to the fitness of the previous position to determine whether that position is used for the next iteration. However, as the dimensionality increases, the algorithm may face a decrease in the diversity of the population at a later stage of the iteration, which increases the risk of falling into a local optimum. This diversity reduction directly affects the algorithm’s convergence speed and the final solution’s accuracy. At the same time, it is clear from the descriptions in “Sine chaotic mapping” and “Opposition-based Learning” that Sine chaotic mappings are random and can perform searches globally. Oppositional learning can increase the diversity of the population and speed up the algorithm’s convergence.

Therefore, this paper proposes a strategy combining Sine chaos mapping and oppositional learning. Firstly, the goal is to reduce the mutual interference between dimensions. Secondly, it will increase the diversity of the algorithm’s search positions and help the algorithm expand the exploration area so that the algorithm gains the ability to get rid of local extremes. Its computational model is:

15 $$\begin{aligned} \overline{x_i^j\mathrm{{ }}} = lb + \left( {ub - x_i^j} \right) \cdot S\left( {x_i^j} \right) \end{aligned}$$

From Eq. (15), compared with general opposition learning, this paper uses Sine opposition learning to perturb the ChoA algorithm to enhance population diversity to increase the likelihood of the algorithm jumping out of the local optimum and, to a certain extent, reduce the likelihood of the algorithm falling into the local optimum, thus improving the optimization efficiency of the algorithm.

Although a reverse solution is generated by Eq. 15, this reverse solution is not necessarily better than the original solution. Therefore, a greedy selection strategy is introduced to choose whether or not to replace the original solution with the reverse solution, i.e. the replacement is made only if the reverse solution has a better fitness value. This approach allows the best position to be introduced into the next iteration with the following computational model:

16 $$\begin{aligned} {X_i}\left( {t + 1} \right) = \left\{ {\begin{array}{*{20}{c}} {{X_i}\left( {t + 1} \right) ,f\left( {\overline{{X_i}} \left( {t + 1} \right) } \right) > f\left( {{X_i}\left( {t + 1} \right) } \right) }\\ {\overline{{X_i}} \left( {t + 1} \right) ,f\left( {\overline{{X_i}} \left( t \right) } \right) \le f\left( {{X_i}\left( {t + 1} \right) } \right) } \end{array}} \right. \end{aligned}$$

Through Eqs. (15) and (16), it can be seen that the Sine dimension-by-dimension opposition learning strategy can be used by generating opposition solutions far from the local extrema when the algorithm falls into a local optimum. The greedy strategy selects the individual with better fitness among the original and inverse solutions, thus generating chimpanzee individuals with better positions. This effectively avoids the decline of population diversity in the late iterations and enhances the algorithm’s global optimality-finding ability. At the same time, a progressively smaller search space can be obtained through the dynamic boundary search mode employed by Sine’s dimension-by-dimension opposition learning. This approach can facilitate the evolution of the CHoA algorithm towards the target position according to different requirements during the iterative process, allowing the algorithm to obtain a better convergence rate.

## Description of SOSCHoA algorithm

### SOSCHoA implementation step

Through the above description, this paper combines the social coevolution strategy, chaotic mapping theory, and the dimension-by-dimension opposition learning strategy to optimize the optimization seeking efficiency and improve the algorithm’s stability to expect better optimization results during each iteration. Therefore, combining the above improvement methods, the SOSCHoA algorithm pseudo-code is given below, with the following steps:

**Figure:**
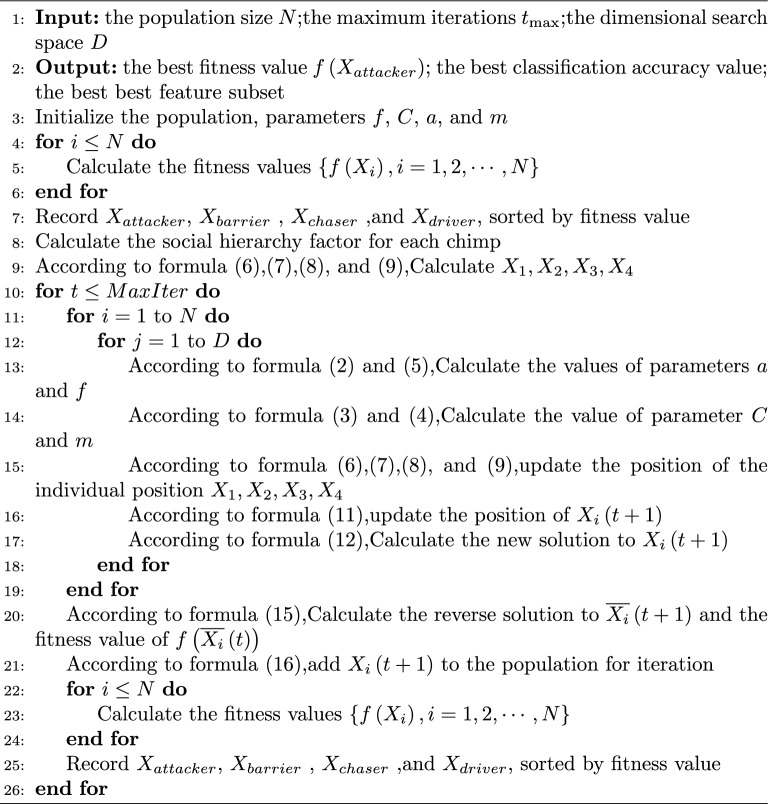
**Algorithm 1** SOSCHoA: the social coevolution and Sine chaotic opposition learning chimp optimization algorithm

Compared with the basic CHoA algorithm, the SOSCHoA algorithm has the following features:

- the SOSCHoA algorithm does not change the framework of the basic CHoA algorithm but only introduces new operators;
- the SOSCHoA algorithm updates the attack prey position through a social coevolution strategy to enhance the local exploration ability;
- the current optimal individual performs a dimension-by-dimension Sine chaos-based opposition learning strategy, enhancing the diversity of the population and reducing the probability of the algorithm falling into a local optimum;
- through a greedy mechanism, allowing the target location to lead in obtaining the global optimal solution.

### Proof of convergence of the SOSCHoA algorithm

Similar to the convergence analysis of most metaheuristic algorithms, we use the deterministic derivation of the SOSCHoA algorithm to analyze its convergence. It is important to note that the convergence proof does not necessarily guarantee that the algorithm converges to the global optimal solution. Since the CHoA algorithm is an intelligent population algorithm, the following theorem follows.

#### Theorem 1

If the CHoA algorithm based on general opposite learning converges, then the SOSCHoA algorithm is also convergent.

#### Proof

let ${X_i}\left( t \right)$ and $\overline{{X_i}} \left( t \right)$ be the current and opposing solutions in generation *t*. $x_i^j\left( t \right)$ and $\overline{x_i^j} \left( t \right)$ are the values of ${X_i}\left( t \right)$ and $\overline{{X_i}} \left( t \right)$ in the *j* dimension, respectively, and the complete solution to the problem is ${x^*}$, which by the conditions in Theorem 1 has for the solution $x_i^j\left( t \right)$ in the *t* generation of the population:

17 $$\begin{aligned} \mathop {\lim }\limits _{t \rightarrow \infty } x_i^j\left( t \right) = x_j^* \end{aligned}$$

Since,$l{b_j}\left( t \right) = \min \left( {x_i^j\left( t \right) } \right)$,$u{b_j}\left( t \right) = \max \left( {x_i^j\left( t \right) } \right)$, it follows:

18 $$\begin{aligned} \mathop {\lim }\limits _{t \rightarrow \infty } l{b_j}\left( t \right) = \mathop {\lim }\limits _{t \rightarrow \infty } u{b_j}\left( t \right) = x_j^* \end{aligned}$$

At *t* generation, the current opposing solution generated by the Sine chaotic opposite learning strategy shown in Eq. (19) is:

19 $$\begin{aligned} \overline{x_i^j\left( t \right) \mathrm{{ }}} = l{b_j}\left( t \right) + \left( {u{b_j}\left( t \right) - x_i^j\left( t \right) } \right) \cdot S \left( {x_i^j\left( t \right) } \right) \end{aligned}$$

When $t \rightarrow \infty$ , from Eq. (19):

20 $$\begin{aligned} \begin{array}{l} \mathop {\lim }\limits _{t \rightarrow \infty } \overline{x_i^j\left( t \right) \mathrm{{ }}} = \mathop {\lim }\limits _{t \rightarrow \infty } l{b_j}\left( t \right) + \mathop {\lim }\limits _{t \rightarrow \infty } \left( {u{b_j}\left( t \right) - x_i^j\left( t \right) } \right) \cdot \mathop {\lim }\limits _{t \rightarrow \infty } S\left( {x_i^j\left( t \right) } \right) \\ \quad = x_j^* + \left( {x_j^* - x_j^*} \right) \cdot \mathop {\lim }\limits _{t \rightarrow \infty } S\left( {x_i^j\left( t \right) } \right) \\ \quad = x_j^* \end{array} \end{aligned}$$

From Eq. (20), when $x_i^j\left( t \right)$ converges to $x_j^*$, the dyadic solution based on Sine chaotic opposition learning strategy also converges to $x_j^*$. Therefore, if the CHoA algorithm based on the general dyadic solution converges, the SOSCHoA algorithm also converges. $\square$

### Time complexity analysis of the SOSCHoA algorithm

The time complexity indirectly reflects the algorithm’s convergence speed. In the CHoA algorithm, the time required to initialize the parameters (population size *N*, *D* search space dimension, *a*, *m*, *f* coefficients, etc.) is assumed to ${\alpha _1}$, the time required to update the positions of other chimpanzee individuals in the population in each dimension according to Eq. (11) is ${\alpha _2}$ and the time required to solve the target fitness function is $f\left( D \right)$, then the time complexity of ChOA is

21 $$\begin{aligned} {T_1}\left( D \right) = O\left( {{\alpha _1} + N\left( {{\alpha _2}D + f\left( D \right) } \right) } \right) = O\left( {D + f\left( D \right) } \right) \end{aligned}$$

In the SOSCHoA algorithm, the time required to initialize the parameters is consistent with the standard ChOA. In the loop phase of the algorithm, let the time required to execute the social symbiosis strategy of ${\alpha _3}$, let the time required to execute the dimension-by-dimensional Sine chaotic opposition learning strategy of ${\alpha _4}$, and the time required to execute the greedy mechanism of ${\alpha _5}$, then the time complexity of SOSCHoA is

22 $$\begin{aligned} {T_2}\left( D \right) = O\left( {{\alpha _1} + {\alpha _5} + N\left( {{\alpha _2}D + {\alpha _3} + {\alpha _4}D + f\left( D \right) } \right) } \right) = O\left( {D + f\left( D \right) } \right) \end{aligned}$$

The SOSCHoA proposed in this paper is consistent with the basic ChOA time complexity.

23 $$\begin{aligned} {T_1}\left( D \right) = {T_2}\left( D \right) = O\left( {D + f\left( D \right) } \right) \end{aligned}$$

In summary, the improvement strategy proposed in this paper for ChOA does not increase the complexity of the time.

### SOSCHoA based feature selection

The feature selection problem for high-dimensional datasets is a binary optimization problem^34^; the solution space is limited to $\left\{ {0,1} \right\}$. For SOSCHoA, it is first necessary to convert continuous optimization values to binary. A feature selection solution can be represented as a searching individual in the SOSCHoA algorithm; the individual dimension is represented as the number of features in the original dataset, and the individual $x_i^j \in \left\{ {0,1} \right\}$. The coding rules are: When $x_i^j = 1$, feature *j* in individual *i* was selected; when $x_i^j = 0$, it means that feature *j* in individual *i* was not selected. For example, Table 2 represents a feature selection solution with an individual dimension of 9, corresponding to an original dataset with nine feature attributes. Of these, $x_i^1 = x_i^2 = x_i^4 = x_i^7 = x_i^8 = 1$ indicates that individual *i* selected features 1, 2, 4, 7 and 8 in the optimal feature subset solution. $x_i^3 = x_i^5 = x_i^6 = x_i^9 = 0$ , this indicates that individual *i* selected features 3, 5, 6, 9 not selected in the optimal feature subset solution. The classifier will use features 1, 2, 4, 7, and 8 as classification data^60^.

**Table 2:** Feature selection solution.

Feature	1	2	3	4	5	6	7	8	9
Location	1	1	0	1	0	0	1	1	0

At the same time, SOSCHoA converts the continuous optimized form to binary form using a conversion function with the following specific functional equation:

24 $$\begin{aligned} x_i^j = \left\{ {\begin{array}{*{20}{c}} {0,x_i^j < 0.5}\\ {1,x_i^j > 0.5} \end{array}} \right. \end{aligned}$$

Where the value of the position in feature *j* in individual *i* is $$x_i^j $$.

At the same time, the feature selection problem for a dataset is a multi-objective optimization problem, requiring the maximum possible data classification accuracy while minimizing the number of features selected. To balance the number of features selected (minimization) and the classification accuracy (maximization), the fitness function is defined as:

25 $$\begin{aligned} f\left( {{X_i}} \right) = \alpha \cdot {\gamma _R}\left( D \right) + \beta \cdot \left( {\frac{{\left| {Selected} \right| }}{{\left| {ALL} \right| }}} \right) \end{aligned}$$

From Eq. (25), ${\gamma _R}\left( D \right)$ denotes the classification error rate (in this paper, the K-Nearest Neighbor (KNN, k=5) algorithm is used to evaluate the classification accuracy of the selected feature subset), $\left| {Selected} \right|$ denotes the number of selected feature sets, and $\left| {ALL} \right|$ denotes the number of original feature sets. $\alpha$ denotes the weighting factor,$\alpha \in \left[ {0,1} \right]$,$\beta = 1 - \alpha$ . Since Eq. (25) plays a large role in the SOSCHoA algorithm searching for the optimal feature subset, it is set to 0.99.

## Experimental validation and analysis

To verify the degradation and classification performance improvement of SOSCHoA for high-dimensional classification data. This section conducts a series of comparison experiments, and the detailed description of the high-dimensional classification dataset used is shown in Table 3. The settings of the comparison algorithms used are presented in Table 4. Second, the classification performance is analyzed, and the number of features in SOSCHoA is investigated. Third, experimental results on classification performance, number of features, and running time are analyzed and evaluated for SOSCHoA versus other heuristic algorithms. Finally, the convergence performance of the compared algorithms and the Wilcoxon rank sum test is verified.

### Description of the experimental dataset

The experimental datasets were selected from the internationally well-known ASU high-dimensional dataset (https://jundongl.github.io/scikit-feature/datasets.html). Table 3 briefly describes these datasets, with the number of samples ranging from 62 to 210, the number of features ranging from 325 to 22,283, and the number of class labels ranging from 2 to 11. When the number of class labels is two categories, it is considered dichotomous. When the number of class labels is more significant than two classes, it is considered multiclassification.

**Table 3:** High-dimensional dataset.

No.	Name of dataset	Number of samples	Number of features	Number of classification labels
1	Lung_discrete	73	325	7
2	Colon	62	2000	2
3	warpAR10P	130	2400	10
4	warpPIE10P	210	2420	10
5	Lung	203	3312	5
6	Lymphoma	96	4026	9
7	Leukemia_1	72	5327	3
8	ALLAML	72	7129	2
9	Carcinom	174	9182	11
10	nci9	60	9712	9
11	Lung_Cancer	203	12,600	5
12	GLI-85	85	22,283	2

### Experimental settings

**Figure 1:**
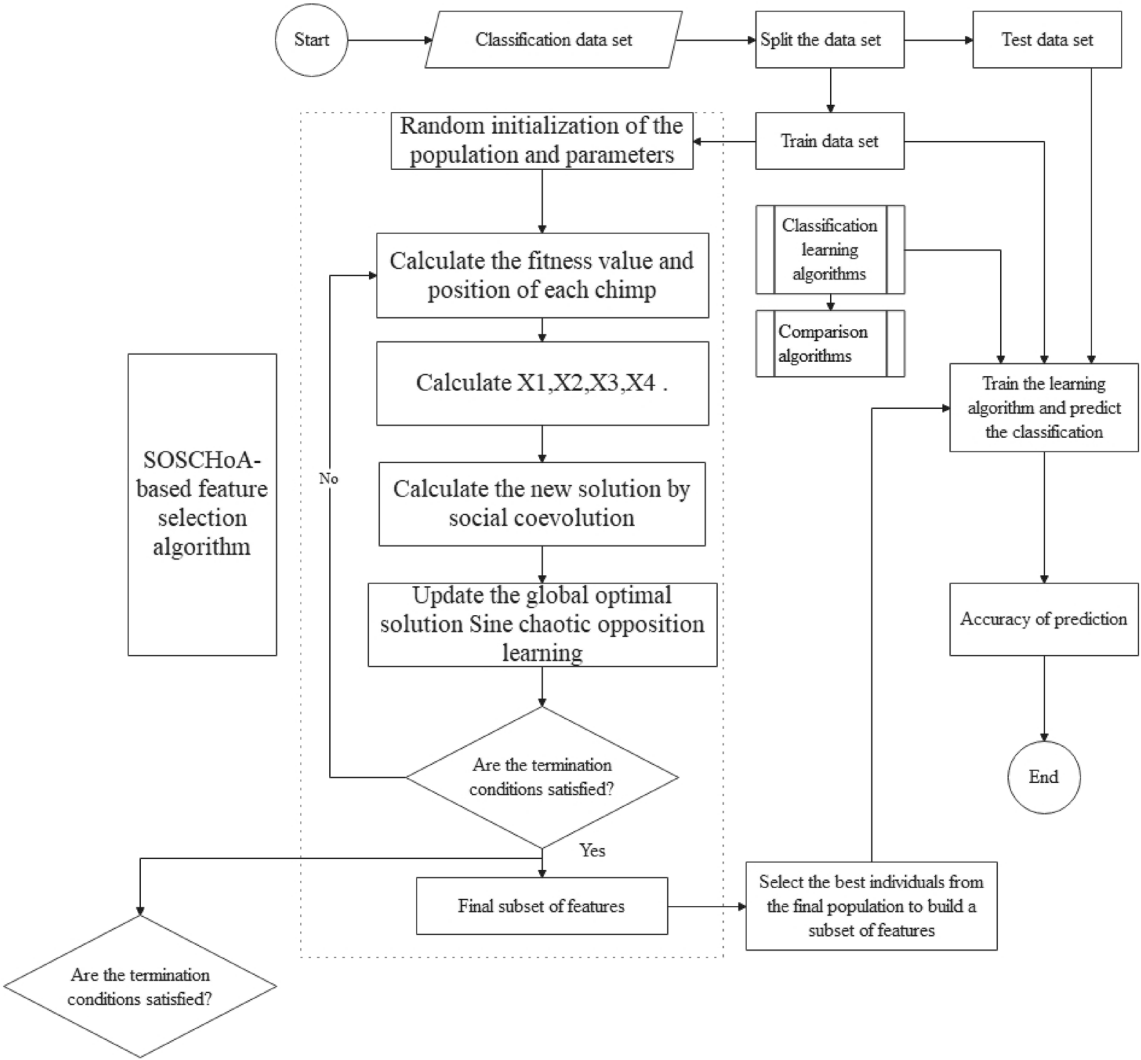
Experimental framework.

To evaluate the impact of the proposed strategy mechanism on the classification performance of high-dimensional microarray data during feature selection, three sets of comparison experiments were designed as follows. In the first set of comparison experiments, the classification performance of SOSCHoA was compared with that of the CHoA algorithm^14^ and the DLFCHOA algorithm^17^. In the second set of comparison experiments, SOSCHoA was compared with PIL-BOA^18^, BBOA^19^, LMRAOA^20^, VGHHO^21^ of different opposing learning element heuristics for comparison of fitness values and classification performance. In the third set of comparison experiments, SOSCHoA was compared with FA^22^, FPA^10^, WOA^12^, HHO^13^, MRFO^23^ for comparison of fitness values and classification performance. The experimental framework is shown in Fig. 1.

Figure 1 shows that SOSCHoA is run on the training dataset to generate a subset of candidate features. Secondly, the training and test sets are transformed into new training and test sets by removing unselected features. Finally, the test dataset is fed into the classifier to verify the classification performance of the selected feature subset against the feature subset selected by the comparison algorithm.

During the experiments, 70% of the samples were randomly selected as training data and 30% of the samples were used as test data. For each test dataset, the experiment was executed *M* times (its value was set to 30) to evaluate the feature selection performance of each method, was the maximum number of iterations the algorithm was run (its value was set to 100), and ${t_{\max }}$ denoted the number of current iterations. The population was put to a uniform 10 to reduce computational costs and maintain search efficiency.

In this experiment, KNN was chosen as the learning algorithm to construct the classifier, and K was set to 5. To verify the optimization effect of the proposed method in the feature selection process, the average classification accuracy, the average number of features selected, the average fitness value, and the standard deviation were used to evaluate the algorithm’s performance, as shown in Eqs. (26)–(31). In addition, a statistical significance test, namely the non-parametric Wilcoxon rank sum test, was performed, and the significance level in the statistical significance test was chosen to be 0.05. All experiments in this paper were conducted in the same environment to ensure fairness in comparing results. The experimental environment for this paper was an Intel-i7 processor with 16GB of RAM and a Windows 10 (64-bit) operating system for the simulation of the proposed algorithm. The algorithm writing simulation software is Python 3.7.0 (https://www.python.org/downloads/) and PyCharm Community Edition2024 (https://www.jetbrains.com/pycharm/download/?section=windows). Table 4 shows the individual algorithm pre-set parameters.

**Table 4:** Comparison algorithm parameter settings.

Algorithm	Population size	Parameter values
CHoA	10	${r_1} \in \left[ {0,1} \right] ,{r_2} \in \left[ {0,1} \right] ,f \in \left( {0,2.5} \right) ,u \in \left[ {0,1} \right]$
DLFCHOA	10	${r_1} \in \left[ {0,1} \right] ,{r_2} \in \left[ {0,1} \right] ,f \in \left( {0,2.5} \right) ,u \in \left[ {0,1} \right]$
SOSCHoA	10	${r_1} \in \left[ {0,1} \right] ,{r_2} \in \left[ {0,1} \right] ,{r_3} \in \left[ {0,1} \right] ,f \in \left( {0,2.5} \right) ,u \in \left[ {0,1} \right]$
PIL-BOA	10	$$\begin{array}{l} \mathrm{{c = 0}}\mathrm{{.01, p = 0}}\mathrm{{.7, a = 0}}\mathrm{{.1,k = 0}}\mathrm{{.5, }}\eta = 10\\ \mu = 0.4,\mathrm{{wmax = 0}}\mathrm{{.9, wmin = 0}}\mathrm{{.2}} \end{array}$$
BBOA	10	$\mathrm{{b = 0}}\mathrm{{.5, wmax = 0}}\mathrm{{.9, wmin = 0}}\mathrm{{.1}}$
LMRAOA	10	$\mathrm{{Mu = 0}}\mathrm{{.499,Alpha = 5,MOP\_Max = 1,MOP\_Min = 0}}\mathrm{{.2}}$
VGHHO	10	$$\begin{array}{l} \mathrm{{c3 = c4 = 2, winitial = 1, wend = 0, }}\\ \mathrm{{Emax = 2, Emin = 0, k = 5, n = 5}} \end{array}$$
FA	10	$\mathrm{{alpha = 1,beta = 1,gamma = 1,theta = 0}}\mathrm{{.97}}$
FPA	10	$P\; = \mathrm{{ }}0.8$
WOA	10	$b = 1$
HHO	10	$beta = 1.5$
MRFO	10	${r_1} \in \left[ {0,1} \right] ,\beta = 2{e^{{r_1}\left( {\left( {MaxIter - t + 1} \right) /MaxIter} \right) }}\sin \left( {2\pi {r_1}} \right)$

The average classification accuracy represents the average of the classification accuracy of the selected feature set, where $acc\left( i \right)$ is the *i*-th classification accuracy and is calculated as follows^61^.

26 $$\begin{aligned} AccMean = \frac{1}{M}\sum \limits _{i = 1}^M {acc\left( i \right) } \end{aligned}$$

The maximum classification accuracy represents the classification accuracy of the selected set of features, calculated as follows.

27 $$\begin{aligned} MaxAcc = \max \left\{ {acc\left( 1 \right) ,acc\left( 2 \right) , \cdots ,acc\left( i \right) } \right\} \end{aligned}$$

The standard deviation of the classification accuracy represents the change in the classification accuracy obtained after running the algorithm and is calculated as follows.

28 $$\begin{aligned} SD = \frac{1}{M}\sum \limits _{i = 1}^M {{{\left( {acc\left( i \right) - AccMean} \right) }^2}} \end{aligned}$$

The average number of features selected describes the average classification accuracy of the selected feature set, where the number of features selected for the *i*-th time is calculated as follows.

29 $$\begin{aligned} NumMean = \frac{1}{M}\sum \limits _{i = 1}^M {number\left( i \right) } \end{aligned}$$

The mean fitness value is calculated as the average of the mean fitness values of the solutions obtained, where the *i*-th fitness value is calculated as follows.

30 $$\begin{aligned} FitMean = \frac{1}{M}\sum \limits _{i = 1}^M {fitness\left( i \right) } \end{aligned}$$

The standard deviation of the fitness value represents the variation in the optimal solution obtained after running the algorithm and is calculated as follows.

31 $$\begin{aligned} Std = \frac{1}{M}\sum \limits _{i = 1}^M {{{\left( {fitness\left( i \right) - fitMean} \right) }^2}} \end{aligned}$$

### Discussion and results

#### Analysis of classification performance versus number of features in SOSCHoA

**Figure 2:**
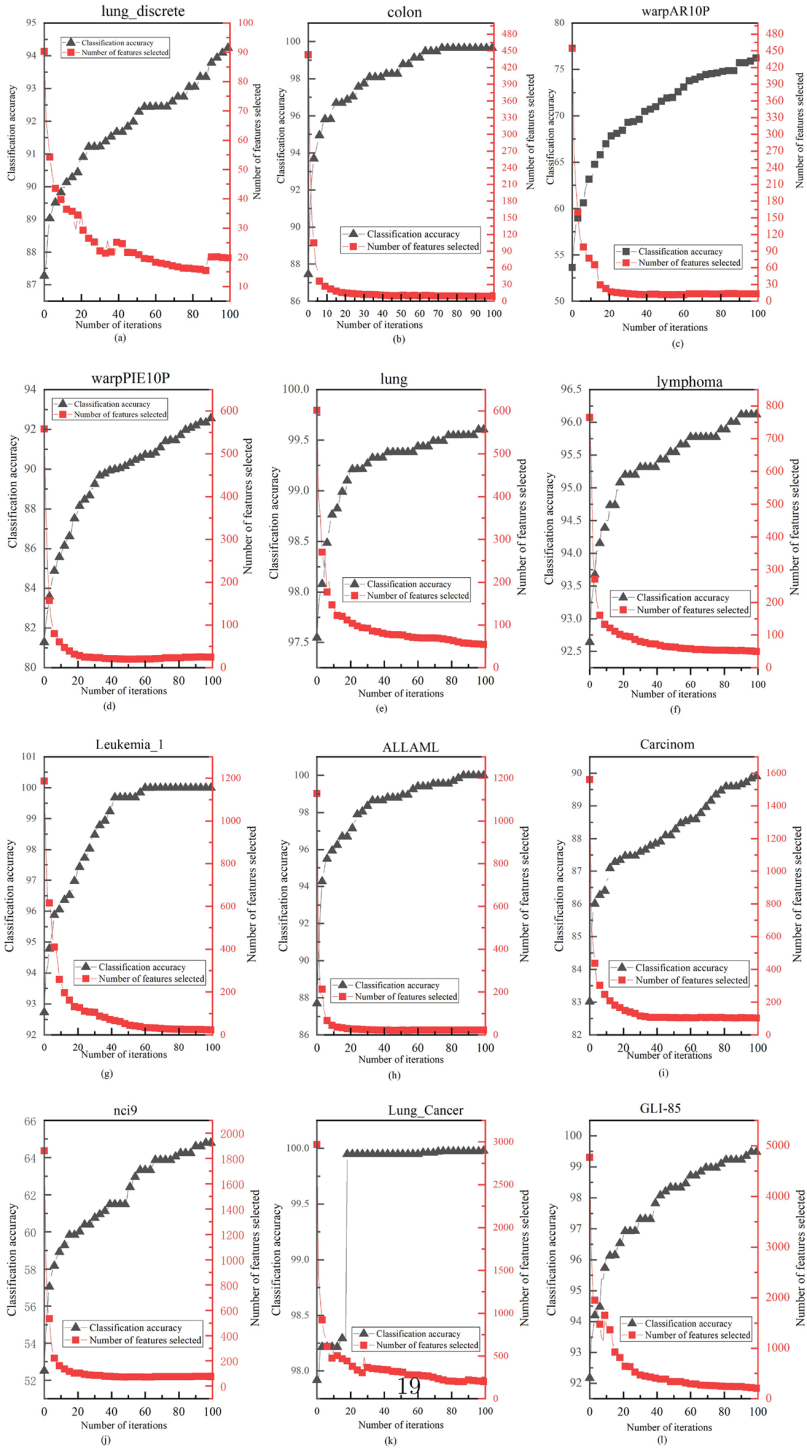
Variation of SOSCHoA classification accuracy versus the number of selected features.

A satisfactory feature selection method should be efficient in reducing the number of features and selecting features relevant to classification accuracy. Figure 2 depicts the variation process of SOSCHoA in terms of classification accuracy and the number of features selected with the increasing number of iterations during the feature selection process.

As seen from Fig. 2, a similar variation trend is shown on different test datasets, i.e. the accuracy of the classifier gradually increases after removing irrelevant or redundant features to the class labels. This indicates that as long as the selected feature subset contains sufficient information, better classification performance will be obtained than using all features. This suggests that SOSCHoA can effectively remove irrelevant or redundant features while improving classification accuracy. Furthermore, a comparative analysis of Table 3 and Fig. 2 shows that the proposed SOSCHoA selects between 0.31% and 6.09% of the original number of features and has significantly reduced the number of features in the original set.

#### SOSCHoA and CHoA diversity analysis

Maintaining diversity in algorithms can yield several benefits, such as increasing the search space, improving algorithm performance and robustness, and avoiding premature convergence.

**Figure 3:**
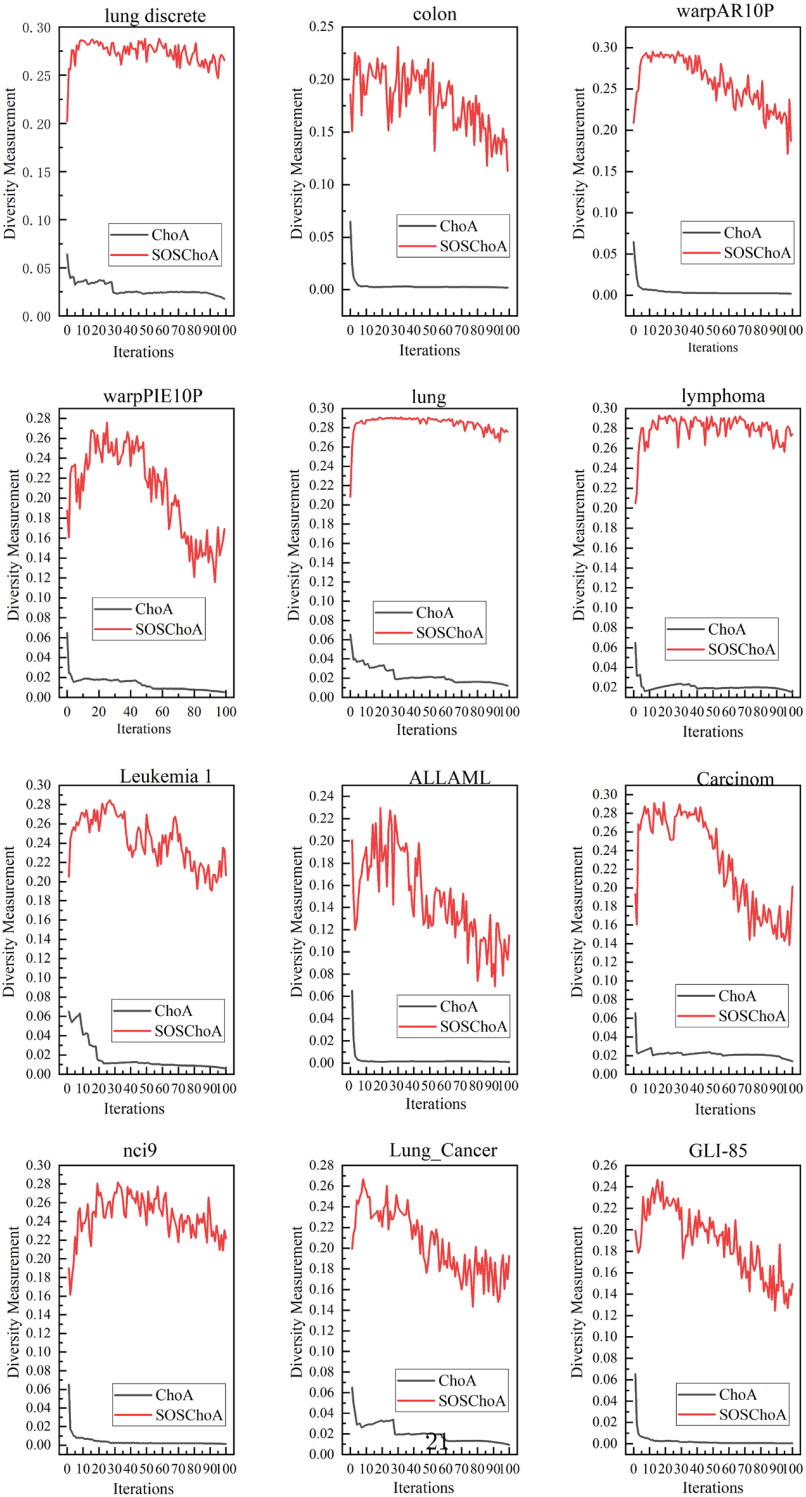
Diversity in SOSCHoA and CHoA.

According to Fig. 3, we measured the diversity of the SOSCHoA and CHoA algorithms during the iteration period. The results show that the SOSCHoA algorithm has more robust diversity than the CHoA algorithm, as derived from the experiments on 12 datasets. This indicates that the SOSCHoA algorithm can effectively improve the interaction and communication between individuals and accelerate the speed of information dissemination, thus improving the efficiency and effectiveness of group collaboration. In addition, the SOSCHoA algorithm can help the group jump out of the local optimal solution and search for the global optimal solution further.

#### Comparison of SOSCHoA with CHoA and DLFCHOA classification performance

In Table 5, AccMean (%), maxAcc (%), and SD denote the average classification accuracy, best classification accuracy, and standard deviation for each algorithm over 30 independent runs on each classification dataset. In Table 6, d and time(/s) denote the average number of features selected and the average running time for each algorithm over 30 independent runs.

**Table 5:** Comparison of the classification performance of SOSCHoA with CHoA and DLFCHOA.

Datasets	DLFCHOA			CHoA			SOSCHoA		
	AccMean (%)	SD	MaxAcc (%)	AccMean (%)	SD	MaxAcc (%)	AccMean (%)	SD	MaxAcc (%)
Lung_discrete	92.58	3.22E−02	100	88.48	2.81E−02	95.45	94.24	2.86E−02	100
colon	95.44	4.65E−02	100	91.75	4.00E−02	100	99.65	1.31E−02	100
WarpAR10P	68.29	5.19E−02	79.49	58.72	4.15E−02	69.23	75.98	4.32E−02	87.18
WarpPIE10P	90.48	2.49E−02	95.24	87.04	1.59E−02	92.06	92.49	2.17E−02	96.83
Lung	99.40	7.90E−03	100	98.36	8.50E−03	100	99.62	6.93E−03	100
Lymphoma	93.33	2.34E−02	96.55	89.43	1.80E−02	93.10	96.57	1.17E−02	96.58
Leukemia_1	94.39	3.25E−02	100	91.82	3.40E−02	100	100	0.00E+00	100
ALLAML	96.67	3.88E−02	100	91.36	4.29E−02	100	100	0.00E+00	100
Carcinom	87.67	2.11E−02	90.57	82.52	2.23E−02	88.68	89.81	2.16E−02	94.34
nci9	**73.15**	3.54E−02	77.78	63.33	3.95E−02	72.22	64.44	3.69E−02	77.78
Lung_Cancer	98.03	1.37E−02	100	96.07	1.31E−02	98.36	98.36	8.96E−03	100
GLI-85	96.54	2.69E−02	100	93.46	3.32E−02	100	99.49	1.31E−02	100

As seen from Table 5, SOSCHoA achieves higher average classification accuracy on all test datasets than the CHoA algorithm. Also, compared to the DLFCHOA algorithm, SOSCHoA achieves higher average classification accuracy on all test datasets except for the nci9 dataset. Regarding standard deviation, SOSCHoA is optimal compared to CHoA on all test datasets except warpPIE10P. SOSCHoA is optimal compared to DLFCHOA on all test datasets except on Carcinom. In conclusion, SOSCHoA showed better performance than DLFCHOA and ChoA algorithms in terms of both average classification accuracy and robustness.

**Table 6:** Comparison of the number of selected features and running time (/t) for SOSCHoA with CHoA and DLFCHOA.

Datasets	DLFCHOA		CHoA		SOSCHoA	
	d	Time	d	Time	d	Time
Lung_discrete	11.63	6.98	12.93	4.95	19.8	12.34
Colon	3.77	39.45	2.9	24.8	8.53	56.11
WarpAR10P	6.63	48.7	5.97	30.81	12.97	64.84
WarpPIE10P	38.07	50.68	32.1	32.86	24.93	68.43
Lung	44.6	66.34	55	43.92	54.4	90.63
Lymphoma	27.2	74.59	26	47.16	49.57	104.84
Leukemia_1	3.93	100.39	4.3	63.76	23.47	133.78
ALLAML	4.27	128.77	4.57	81.84	22.07	178.63
Carcinom	187.07	178.6	176.3	116.52	103.17	237.97
nci9	64.13	172.66	39.03	107.91	74.47	234.22
Lung_Cancer	244.63	233.7	175.2	149.76	275	310.66
GLI-85	16.27	400.28	6.53	252.51	207.07	505.85
Average	54.35	125.1	45.07	79.73	66.66	166.53

As can be seen from Table 6, SOSCHoA has the highest average number of features selected among the three algorithms at 66.66, which is 12.31 and 21.59 higher than DLFCHOA and ChoA, respectively. This indicates that SOSCHoA still needs to improve its feature selection capability and optimise the number of selected features.

#### Analysis of CHoA algorithm improvement strategies

The data in Table 3 were selected for classification accuracy and adaptation value experiments to analyse the improved strategies’ impact on the algorithms’ performance. The CHoA algorithm that only employs the social coevolution strategy (SOCHoA) is compared with the CHoA algorithm that escapes the local optimal solution using the Sine chaotic opposing learning strategy (SCHoA). The parameters of the above two algorithms are the same as in “Experimental settings”.

**Table 7:** Comparison of classification accuracy and average fitness value test results of algorithms.

Data name	Classification accuracy			Average fitness values		
	SOSCHoA (%)	SOCHoA (%)	SCHoA (%)	SOSCHoA	SOCHoA	SCHoA
Lung_discrete	94.24	85.00	85.61	5.76E−02	1.49E−01	1.43E−01
colon	99.65	93.86	96.32	3.52E−03	6.08E−02	3.65E−02
warpAR10P	75.98	69.66	70.77	2.38E−01	3.00E−01	2.89E−01
warpPIE10P	92.49	96.19	95.93	7.45E−02	3.78E−02	4.05E−02
lung	99.62	99.34	100.00	3.95E−03	6.59E−03	2.69E−04
lymphoma	96.57	96.21	96.55	3.43E−02	3.76E−02	3.44E−02
Leukemia_1	100	99.85	100.00	4.41E−05	1.51E−03	1.03E−05
ALLAML	100	97.73	99.39	3.10E−05	2.25E−02	6.00E−03
Carcinom	89.81	98.05	97.55	1.01E−01	1.95E−02	2.46E−02
nci9	64.44	69.63	67.22	3.52E−01	3.01E−01	3.25E−01
Lung_Cancer	98.36	99.02	100.00	1.65E−02	9.82E−03	3.78E−04
GLI-85	99.49	96.54	97.31	5.17E−03	3.43E−02	2.67E−02

The comparison results from Table 7 show that the operator’s classification accuracy and average adaptation value using the social Coevolution strategy are significantly better than the SOSCHoA algorithm on the warpPIE10P, Carcinom and nci9 datasets. The operator’s classification accuracy and average adaptation value using the Sine chaotic Opposing learning strategy are significantly better than the SOSCHoA algorithm on the lung and Lung_Cancer datasets. Meanwhile, by combining the results in Tables 5 and 7, it can be seen that SOCHoA and SCHoA classification accuracy and average adaptation value perform poorly on the lung_discrete dataset, which suggests that only adopting the Social Coevolution strategy or only the Sine Chaos Opposing Learning strategy can be of significant help in improving the performance of the CHoA algorithm.

In conclusion, the results of SOSCHoA are better than the two sub-algorithms of SOCHoA and SCHoA. The comparison results show that both improvement strategies play a role in improving the algorithm, and their promotion can be effectively combined without being suppressed by either operator, which confirms the effectiveness of the improvement strategies for the algorithm. Therefore, the SOSCHoA algorithm can improve the CHoA algorithm, strengthen its global investigation and local mining ability, accelerate the convergence speed, eliminate the local optimum, and achieve higher classification accuracy and smaller optimal adaptation value.

#### Analysis of the impact of opposing learning strategies on classification performance

To verify the superiority of SOSCHoA, algorithms with different opposing learning strategies were selected to compare and validate the classification performance of the test data, specifically PIL-BOA, BBOA, LMRAOA, and VGHHO. The algorithms were tested for classification comparison by using the 12 test datasets given in Table 3. Each algorithm was run 30 times to obtain the average classification values, and the comparison results are shown in Table 8.

**Table 8:** Analysis of the running time (/s) and classification accuracy of SOSCHoA with different opposing learning strategy algorithms.

Datasets	PIL-BOA		VGHHO		LMRAOA		BBOA		SOSCHoA	
	AccMean	Time	AccMean	Time	AccMean	Time	AccMean	Time	AccMean	Time
Lung_discrete	90.15%	3.06	88.79%	1.48	91.06%	11.04	91.97%	3.54	94.24%	12.34
Colon	83.33%	5.27	80.53%	2.75	77.37%	35.36	85.96%	5.47	99.65%	56.11
WarpAR10P	54.79%	7.45	51.88%	3.94	51.54%	46.9	58.21%	7.85	75.98%	64.84
WarpPIE10P	87.57%	8.79	85.40%	4.48	87.30%	48.17	89.10%	8.88	92.49%	68.43
Lung	99.73%	11.68	99.56%	6.34	100.00%	64.57	100.00%	11.8	99.62%	90.63
Lymphoma	93.22%	10.09	92.64%	5.6	93.10%	74.68	94.25%	10.59	96.57%	104.84
Leukemia_1	91.21%	13.61	89.39%	7.91	95.00%	102.06	94.39%	14.49	100%	133.78
ALLAML	92.88%	15.47	89.85%	9	89.55%	126.65	95.61%	16.21	100%	178.63
Carcinom	90.75%	27.01	89.94%	14.5	92.33%	179.84	91.32%	26.84	89.81%	237.97
nci9	54.44%	22.22	52.78%	13.05	52.78%	187.73	59.81%	23.29	64.44%	234.22
Lung_Cancer	95.08%	38.52	94.97%	20.67	98.14%	244.59	96.78%	36.82	98.36%	310.66
GLI-85	92.95%	51.4	91.15%	29.9	92.69%	426.05	95.90%	52.76	99.49%	505.85

From the results in Table 8, the classification performance of SOSCHOA was only better than that of VGHHO on the lung. Regarding carcinoma, the classification performance of SOSCHOA was the worst. For all other datasets, the classification performance of SOSCHOA was better than that of the other metaheuristics. This indicates that SOSCHOA has a significant advantage over the different algorithms in terms of classification performance. Also, the running time of the SOSCHOA algorithm is well within the acceptable range.

#### SOSCHoA is compared with other algorithms for classification performance

To further demonstrate the effectiveness of the SOSCHoA algorithm, it was compared with the five different heuristic optimization algorithms. Table 9 shows the average classification accuracy of these five algorithms. Table 10 indicates the number of features selected for these five algorithms. Table 11 shows the average running time of these five algorithms.

**Table 9:** Average classification accuracy performance of SOSCHoA and the other four heuristic optimization algorithms.

Datasets	FA (%)	FPA (%)	WOA (%)	MRFO (%)	HHO (%)	SOSCHoA (%)
Lung_discrete	91.06	90.15	89.55	81.21	82.73	94.24
Colon	88.42	84.91	91.58	80.88	91.23	99.65
WarpAR10P	52.65	51.11	55.47	54.27	65.21	75.98
WarpPIE10P	95.24	95.24	95.34	90.00	90.63	92.49
Lung	100.00	98.91	99.34	97.54	97.65	99.62
Lymphoma	89.89	89.89	93.56	95.40	96.09	96.57
Leukemia_1	100.00	99.85	99.39	90.45	92.42	100
ALLAML	77.27	77.12	87.12	86.21	95.00	100
Carcinom	86.79	85.28	87.30	89.25	89.94	89.81
nci9	31.48	29.07	54.26	54.44	66.30	64.44
Lung_Cancer	98.52	97.49	98.36	95.36	95.96	98.36
GLI-85	96.79	95.51	97.05	93.97	94.87	99.49
A verage	84.01	82.88	87.36	84.08	88.17	92.55

Table 9 shows that on the warpPIE10P dataset, WOA classification accuracy was the best, and SOSCHoA classification accuracy ranked third. On the lung and Lung_Cancer datasets, FA classification accuracy was the best, and SOSCHoA classification accuracy ranked second. For the Carcinom and nci9 datasets, HHO classification accuracy was the best, and SOSCHoA classification accuracy ranked second. SOSCHOA’s classification performance for all other datasets was better than that of the other metaheuristics. This indicates that SOSCHOA has a significant advantage over the different algorithms in terms of classification performance.

**Table 10:** Average number of features selected for SOSCHoA and other heuristic optimization algorithms.

Datasets	FA	FPA	WOA	MRFO	HHO	SOSCHoA
Lung_discrete	146.13	151.97	52.6	48.37	32.1	19.8
Colon	973.2	968.73	78.3	374.93	32.87	8.53
WarpAR10P	1178.83	1170.73	260.13	383.87	71.9	12.97
WarpPIE10P	1144.3	1160.83	408.83	487.13	272.87	24.93
Lung	1621.03	1590	474.43	729.03	382.03	54.4
Lymphoma	1919.97	1930.03	133.7	619.6	527.4	49.57
Leukemia_1	2577.23	2596.1	1491.2	1913.77	869	23.47
ALLAML	84.5	3461.13	3489.63	35.37	942.67	22.07
Carcinom	4536.13	4499.7	1277.73	2854.17	1651.63	103.17
nci9	4801.27	4794.13	119.87	1425.47	156.23	74.47
Lung_Cancer	6245.93	6232.23	1591.27	3609.27	1714.07	275
GLI-85	11030.27	11017.87	3056.17	5134.87	2608.97	207.07
Average	3021.57	3297.79	1036.16	1467.99	771.81	72.95

As seen from Table 10, the number of features selected by SOSCHoA is lower on all test datasets compared to the five algorithms, FA, FPA, WOA, MRFO, and HHO. From Tables 9 and 10, it can be seen that the SOSCHoA algorithm is the most efficient.

**Table 11:** Running time (/s) of SOSCHoA and other kinds of heuristic optimization algorithms.

Datasets	FA	FPA	WOA	MRFO	HHO	SOSCHoA
Lung_discrete	7.85	1.62	1.86	4.24	2.9	12.34
Colon	25.41	4.79	6.23	13.89	7.24	56.11
WarpAR10P	32.06	6.12	7.57	18.08	9.51	64.84
WarpPIE10P	37.24	7.31	8.64	19.98	10.84	68.43
Lung	53.74	10.39	11.77	26.67	13.79	90.63
Lymphoma	50.05	9.55	12.17	26.62	13.7	104.84
Leukemia_1	56.49	10.86	15.13	34.73	17.41	133.78
ALLAML	76.35	14.55	19.21	49.46	23.9	178.63
Carcinom	119.77	23.37	27.56	64.95	34.15	237.97
nci9	107.32	19.92	27.05	56.79	28.58	234.22
Lung_Cancer	165.78	32.52	36.78	88.2	45.18	310.66
GLI-85	238.7	44.5	59.75	129.41	66.52	505.85
Average	80.9	15.46	19.48	44.42	22.81	166.53

As seen from Table 11, the running time of the SOSCHoA algorithm is still relatively long due to the larger search space in high-dimensional data. However, the running time of the SOSCHOA algorithm is well within the acceptable range.

In summary, the SOSCHoA algorithm has a robust search capability and can find a relatively small and high-quality subset of features. Secondly, it shows that the SOSCHoA algorithm can improve the classification accuracy in the selected feature subset. Finally, it also indicates that the chosen feature subset by the SOSCHoA algorithm still has room for further reduction and improvement in classification accuracy. This also provides a feasible study for subsequent research and the design of new innovative mechanisms to eventually reduce the size of the feature subset and further improve the model’s classification performance. In summary, among the six methods, the feature selection method proposed by SOSCHoA can better balance the classification performance and the selected feature subset.

### SOSCHoA compared with other algorithms for convergence

**Figure 4:**
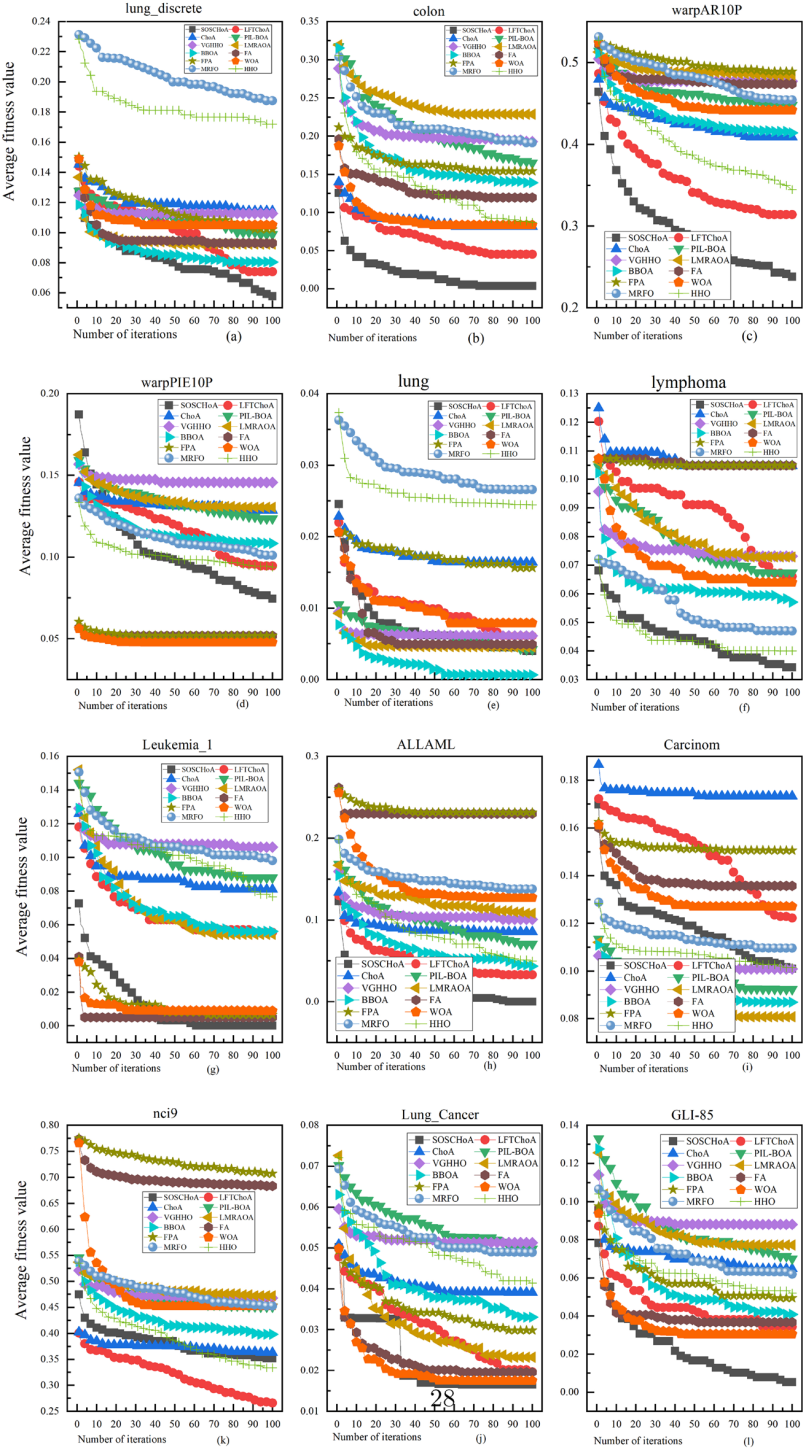
Comparison of the convergence curves of the SOSCHoA algorithm with the other eleven compared algorithms.

Each algorithm was run 30 times independently during the experiments, and the average fitness values in each generation were recorded. Thus, 100 iterations correspond to 100 average fitness values for each algorithm. As the goal of the feature selection process is to minimize the value of the fitness function, the smaller the value of the fitness function, the better the convergence performance of the corresponding algorithm. Figure 4 shows the convergence curves of the SOSCHoA algorithm compared to the other 12 compared algorithms. From Fig. 4, it can be seen that the SOSCHoA algorithm has a faster convergence rate on eight of the 12 test datasets (lung_discrete, colon, warpAR10P, lymphoma, Leukemia_1, ALLAML, Lung_Cancer, and GLI-85). For the remaining four test datasets (warpPIE10P, lung, Carcinom, and nci9), the SOSCHoA algorithm also showed better convergence performance than most comparison algorithms. This further indicates that the mechanism designed in the SOSCHoA algorithm effectively enhances the algorithm’s search capability, thus enabling a higher-quality subset of features to be found in a limited number of iterations. The results in Tables 8 and 9 also demonstrate the effectiveness of the SOSCHoA algorithm for searching in high-dimensional feature spaces.

The results show that the improved mechanism proposed by the SOSCHoA method can effectively improve the classification accuracy and reduce the dimensionality of selected data features in sample data of different dimensions. Secondly, it can perform the feature selection task effectively and successfully and performs better classification in determining features with discriminative solid power.

### Algorithmic stability analysis

**Figure 5:**
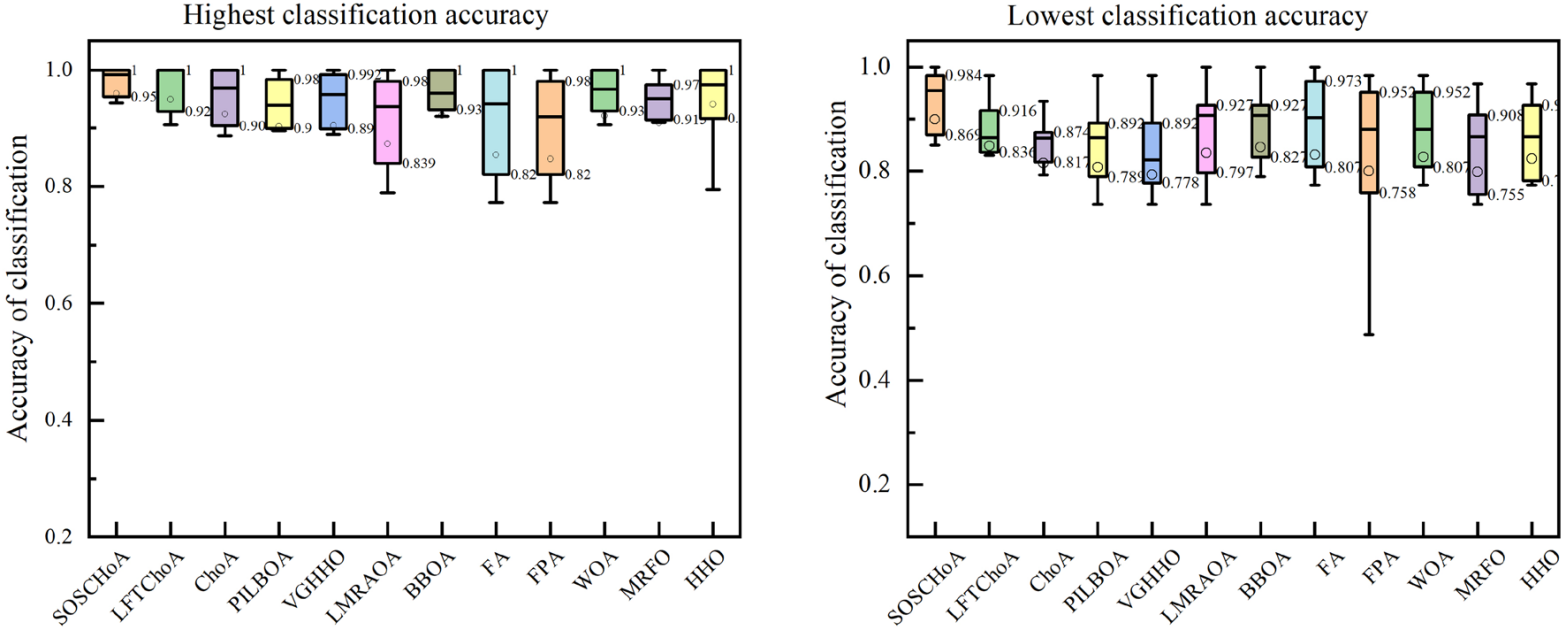
Comparison of the convergence curves of the SOSCHoA algorithm with the other eleven compared algorithms.

To evaluate the stability of SOSCHoA on high-dimensional datasets, this paper conducts 30 experiments using 12 different test datasets. The results of these experiments are shown in Fig. 5, which illustrates the distribution of data with the highest and lowest classification accuracy.

The boxplot in Fig. 5 shows that SOSCHoA performs optimally for the minimum, 25th percentile, median, 75th percentile and maximum values and exhibits good stability without significant fluctuations. These results illustrate that the algorithm can show good performance and robustness on different datasets and has relatively little effect on outliers.

### Wilcoxon rank-sum test

To verify the fairness and stability of the SOSCHoA algorithm, this study used the Wilcoxon rank sum test, a non-parametric statistical test, to compare the median of two independent samples. The test’s null hypothesis (H0) is that there is no significant difference between the two algorithms in terms of performance, while the alternative hypothesis (H1) is that the SOSCHoA algorithm significantly outperforms the comparison algorithm in terms of performance. We set the significance level to 5%, which means that if the p-value is less than 0.05, we will reject the null hypothesis and consider the two algorithms significantly different in performance. Conversely, if the p-value is greater than or equal to 0.05, the two algorithms are considered not significantly different regarding overall search results. When $p < 5\%$, there is a significant difference between the two algorithms compared. When $p \ge 5\%$, the two algorithms compared are identical in their overall search results. Also, SOSCHoA was compared with LFTChoA, ChoA, PIL-BOA, VGHHO, LMRAOA, BBOA, FA, FPA, WOA, MRFO, and HHO, noted as P1, P2, P3, P4, P5, P6, P7, P8, P9, P10 and P11, respectively. Table 10 gives the values calculated in the rank sum test of SOSCHoA against LFTChoA, ChoA, PIL-BOA, VGHHO, LMRAOA, BBOA, FA, FPA, WOA, MRFO, and HHO for the 12 tested data sets.

As can be seen from the analysis in Table 12, the values are much less than 5% in the vast majority of the tested Datasets. Among them, on the warpPIE10P and lymphoma datasets, the search results of the SOSCHOA and HHO algorithms are identical overall. The SOSCHOA and VGHHO algorithms had the same overall optimization results in the lung dataset. In the Leukemia_1 dataset, SOSCHOA and the WOA and MRFO algorithms were found to be identical overall, respectively. These results show that the SOSCHoA algorithm usually provides statistically significant performance improvements. However, we also note that on specific datasets, the performance of SOSCHoA is similar to that of the other algorithms. This may be due to the characteristics of these datasets or the inherent advantages of different algorithms in dealing with specific problems.

**Table 12:** Wilcoxon rank-sum test p-values.

Datasets	P1	P2	P3	P4	P5	P6
Lung_discrete	3.88E−18	3.88E−18	3.89E−18	4.65E−18	3.17E−16	3.49E−13
Colon	3.35E−18	3.02E−18	3.86E−18	3.54E−18	3.71E−18	3.71E−18
WarpAR10P	3.84E−18	3.86E−18	3.89E−18	3.88E−18	3.88E−18	3.88E−18
WarpPIE10P	7.06E−11	1.02E−14	7.76E−17	4.32E−17	9.50E−17	2.64E−10
Lung	5.48E−16	4.16E−18	3.64E−04	7.47E−01	5.12E−14	3.86E−18
Lymphoma	4.16E−23	4.16E−23	4.16E−23	4.16E−23	4.16E−23	4.16E−23
Leukemia_1	3.60E−18	3.48E−18	3.81E−18	3.66E−18	3.48E−18	3.78E−18
ALLAML	3.28E−18	3.42E−18	3.82E−18	3.68E−18	3.80E−18	3.80E−18
Carcinom	3.87E−18	3.86E−18	3.88E−18	3.86E−18	3.84E−18	3.88E−18
nci9	3.87E−18	1.78E−04	3.86E−18	3.84E−18	3.80E−18	3.78E−18
Lung_Cancer	1.40E−20	2.10E−21	4.16E−23	4.16E−23	1.90E−17	4.16E−23
GLI-85	3.85E−18	3.76E−18	3.83E−18	3.83E−18	3.84E−18	3.80E−18

Datasets	P7	P8	P9	P10	P11	
Lung_discrete	6.10E−18	3.89E−18	3.88E−18	3.88E−18	3.88E−18	
Colon	3.27E−18	3.60E−18	3.06E−18	3.86E−18	3.84E−18	
WarpAR10P	3.88E−18	3.87E−18	3.88E−18	3.89E−18	3.88E−18	
WarpPIE10P	3.87E−18	3.87E−18	3.87E−18	2.00E−06	8.50E−01	
Lung	1.34E−10	4.49E−18	7.12E−16	3.81E−18	3.87E−18	
Lymphoma	4.16E−23	4.16E−23	4.16E−23	4.16E−23	4.84E−01	
Leukemia_1	3.60E−02	7.80E−01	9.53E−01	3.88E−18	3.88E−18	
ALLAML	3.63E−18	3.70E−18	3.49E−18	3.87E−18	3.78E−18	
Carcinom	4.09E−18	3.98E−18	1.01E−17	5.00E−06	6.11E−18	
nci9	3.74E−18	3.77E−18	3.79E−18	3.84E−18	1.50E−05	
Lung_Cancer	2.16E−04	1.40E−20	2.16E−04	4.16E−23	4.16E−23	
GLI-85	3.17E−17	3.80E−18	1.66E−17	3.88E−18	3.86E−18	

## Conclusion

When dealing with high-dimensional classification data, the complex interactions between features pose higher challenges to feature selection algorithms. The traditional CHoA has limitations in fast convergence and accurate optimization search, and it is difficult to identify and eliminate irrelevant and redundant features efficiently. To overcome these limitations and improve the global search capability and convergence efficiency of the algorithm, after an in-depth study of the core mechanism of CHoA, this paper proposes a new algorithm: Social Coevolution and Sine Chaotic Oppositional Learning Chimp Optimization Algorithm (SOSCHoA). The improvements of the SOSCHoA algorithm are mainly reflected in the following aspects:

- Introducing the social coevolution strategy, which enhances the information exchange between individuals, extends the search subspace and dynamically adjusts the balance between local exploration and global exploitation.
- Using a sine chaotic opposition learning increases the diversity of the population. It improves the ability of the algorithm to jump out of the local optimum and approach the global optimal solution.
- Experimental results show that SOSCHoA significantly outperforms existing algorithms such as CHoA, DLFCHOA, PIL-BOA, BBOA, VGHHO, FA, FPA, WOA, HHO, and MRFO in terms of convergence rate, classification accuracy, and feature approximation ability. These results confirm the significant advantages of SOSCHoA in improving classification accuracy and reducing the number of features. However, regarding reducing the number of feature dimensions, the SOSCHoA algorithm still needs to catch up on datasets such as warpPIE10P, lung, Carcinom and nci9.

Future research will focus on further optimizing the position update equation and the global exploration mechanism to improve the high-dimensional classification optimization capability of SOSCHoA, especially when dealing with datasets with higher feature dimensions.

## Software tools

PyCharm Community Edition2024 (https://www.jetbrains.com/pycharm/download/?section=windows); Python 3.7.0 (https://www.python.org/downloads/)

## Data Availability

The experimental data set selects the world-famous data set (https://jundongl.github.io/scikit-feature/datasets.html).
